# Prefrontal Hemodynamics in Toddlers at Rest: A Pilot Study of Developmental Variability

**DOI:** 10.3389/fnins.2017.00300

**Published:** 2017-05-30

**Authors:** Afrouz A. Anderson, Elizabeth Smith, Fatima A. Chowdhry, Audrey Thurm, Emma Condy, Lauren Swineford, Stacy S. Manwaring, Franck Amyot, Dennis Matthews, Amir H. Gandjbakhche

**Affiliations:** ^1^Eunice Kennedy Shriver National Institute of Child Health and Human Development, National Institutes of HealthBethesda, MD, United States; ^2^National Institute of Mental Health, National Institutes of HealthBethesda, MD, United States; ^3^Department of Speech and Hearing Sciences, Elson S. Floyd College of Medicine, Washington State UniversitySpokane, WA, United States; ^4^Communication Science and Disorders, University of UtahSalt Lake City, UT, United States; ^5^Center for Neuroscience and Regenerative MedicineRockville, MD, United States; ^6^Department of Neurology, Uniformed Services University of the Health ScienceBethesda, MD, United States; ^7^Department of Neurological Surgery, School of Medicine, University of California, DavisDavis, CA, United States

**Keywords:** Near-infrared spectroscopy, autoregulation, hemodynamics, toddler neuroimaging, cognitive development

## Abstract

Functional near infrared spectroscopy (fNIRS) is a non-invasive functional neuroimaging modality. Although, it is amenable to use in infants and young children, there is a lack of fNIRS research within the toddler age range. In this study, we used fNIRS to measure cerebral hemodynamics in the prefrontal cortex (PFC) in 18–36 months old toddlers (*n* = 29) as part of a longitudinal study that enrolled typically-developing toddlers as well as those “at risk” for language and other delays based on presence of early language delays. In these toddlers, we explored two hemodynamic response indices during periods of rest during which time audiovisual children's programming was presented. First, we investigate Lateralization Index, based on differences in oxy-hemoglobin saturation from left and right prefrontal cortex. Then, we measure oxygenation variability (OV) index, based on variability in oxygen saturation at frequencies attributed to cerebral autoregulation. Preliminary findings show that lower cognitive (including language) abilities are associated with fNIRS measures of both lower OV index and more extreme Lateralization index values. These preliminary findings show the feasibility of using fNIRS in toddlers, including those at risk for developmental delay, and lay the groundwork for future studies.

## Introduction

Functional near infrared spectroscopy (fNIRS) is a non-invasive, affordable, compact instrument for measuring functional brain activity. Compared to fMRI or PET, fNIRS is less susceptible to motion artifacts, making it an appropriate alternative to acquire brain activity data in infants and young children. Multiple studies have used fNIRS to investigate cerebral hemodynamics in both typical infants and infants at risk for neurodevelopmental disorders (e.g., Keehn et al., 2013; Gomez et al., 2014; Lloyd-Fox et al., 2015) as well as in older preschoolers (e.g., Perlman et al., 2016; Li et al., in press). The current study focuses on the more challenging toddler period between 18–36 months of age, when language delay and other developmental problems are first noted, and tools measuring neural correlates of potential delays are needed.

fNIRS uses light in the near infrared range to measure changes in concentration of oxy-hemoglobin (HbO) and deoxy-hemoglobin (Hb) in cortical regions. Since neural activity is associated with increased demand for HbO, changes in light absorption in the near infrared range are reflective of brain activation (Lam et al., 1997; Yodh and Boas, 2003; Boas et al., 2004; Gratton et al., 2005). Therefore, like fMRI, fNIRS infers activation from hemodynamic response. Previous studies have indicated a strong correlation between fNIRS and fMRI signals (Huppert et al., 2006; Sassaroli et al., 2006; Amyot et al., 2012). By capitalizing on its ease of use and localizable cerebral hemodynamic signals, fNIRS can be used to characterize the neural substrates of developmental differences in toddlers.

Language delays in the first three years of life indicate risk for later diagnoses of autism spectrum disorder (ASD), intellectual disability, and specific language impairment (Michelotti et al., 2002). Identifying characteristics related to brain function that accompany language or more general delay can improve our understanding of the neurobiological timeline within which such delays and their sequelae occur. In the current study, we investigate both the feasibility of fNIRS within the toddler years as well as two potential metrics for detection of individual variation in neural activity.

First, we analyze the oxygenation variability (OV) index, a measure of change in hemodynamic response in the frequency range associated with Cerebral Autoregulation (CA). Various physiological mechanisms can result in hemodynamic oscillations at specific frequencies (Obrig et al., 2000; Sassaroli et al., 2012). Specifically, CA maintains cerebral blood flow (CBF) by means of vasoconstriction and vasodilation (vasomotion), and is thus a necessary process for precise regulation of cerebral hemodynamics and circulation. Cerebral autoregulation is related to brain function in typical development (Chiron et al., 1992; Udomphorn et al., 2008; Cipolla, 2009; Kilroy et al., 2011; Anderson et al., 2014) and has been linked to poor developmental and cognitive outcomes in children and adults (Muizelaar et al., 1991; Lam et al., 1997; Udomphorn et al., 2008; Liu et al., 2015; Chernomordik et al., 2016). Spontaneous hemodynamic oscillations at frequencies of <0.1 Hz are known to be associated with cerebral autoregulation in children (Bassan et al., 2005; Wong et al., 2008) and are related to the strength and degree of cerebral autoregulation based on vasomotion (Sassaroli et al., 2012; Kainerstorfer et al., 2015; Liu et al., 2015). Here, using fNIRS, we assess the OV Index (Anderson et al., 2014) to quantify changes in oxygen saturation oscillations in frequencies associated with cerebral autoregulation in toddlers and relate those changes to individual variability in developmental ability.

Additionally, hemispheric lateralization has been a target in the search for early brain markers related to neurodevelopment due to its early presence in infant development and the association of aberrant lateralization with atypical development. Some aspects of language processing are lateralized at birth (Pena et al., 2003; Telkemeyer et al., 2009), and lateralization persists through infancy (Minagawa-Kawai et al., 2011) and into childhood (Sato et al., 2010, 2011; May et al., 2011). The capability that NIRS has for detecting lateralization patterns at birth makes it a promising method for potentially detecting early differences in infants who may be at risk for a neurodevelopmental disorder. In addition, functional lateralization (particularly to linguistic stimuli) varies in individuals with a range of neurodevelopmental concerns, including autism, specific language impairment, and dyslexia (e.g., Whitehouse and Bishop, 2008; Lindell and Hudry, 2013; Nielsen et al., 2014; Xu et al., 2015). Apart from language, lateralization of the Prefrontal Cortex, and its relation to higher cognitive function has also been a focus in the literature (Van Horn et al., 1996; Dumontheil et al., 2008; Christoff, 2009; Kawakubo et al., 2011; Burgess and Wu, 2013) due to its important role in cognitive development in both children and adolescents (Miller and Cohen, 2001; Kwon et al., 2002; Wood and Grafman, 2003; Hare and Casey, 2005; Casey et al., 2008; Tsujimoto, 2008; Boschin et al., 2015). Lateralization differences in the PFC have been noted in both individuals with ASD and adults with mild cognitive impairments (Tamura et al., 2012; Kikuchi et al., 2013; Zhu et al., 2015; Yeung et al., 2016). Therefore, lateralization of the PFC could play an important role in understanding and tracking cognitive development in toddlers.

Therefore, while the fNIRS literature in infants, children, and adults frequently focuses on functional (i.e., event related) changes in oxygenation, here we focus on two alternative metrics that can be used in alert, relaxed toddlers. Specifically, we measure Laterality Index, which captures the percent difference between left and right hemodynamic response. While positive values indicate more leftward activation and negative more rightward, more extreme values (i.e., higher absolute values) indicate more unilateral vs. bilateral patterns of activation. The OV index, quantifies the level of variations in the oxygen saturation signal, where higher values indicate greater variability in oxygenation levels, which is related to dynamics of cerebral hemodynamics.

Similar to other studies in young children, who are less likely to tolerate controlled laboratory stimuli, we measured activity while toddlers underwent a “vanilla baseline” period (Jennings et al., 1992) which consisted of watching and listening to an engaging children's show (Kikuchi et al., 2013; Fekete et al., 2014; Li and Yu, 2016). This method of acquiring usable data while very young children rest has been extensively used to measure brain activity through EEG techniques in various populations, including toddlers at risk for neurodevelopmental problems (Elsabbagh et al., 2009; Tierney et al., 2012). Here, we combine this commonly-used method whereby toddlers are kept alert and calm with audiovisual presentation of children's videos, with fNIRS measurement of both Laterality Index and OV Index. This allows for exploration how prefrontal hemodynamics relate development in toddlers, including those unable to tolerate an absence of stimuli or presence of repetitive, controlled stimuli. In order to improve tolerance and data quality in toddlers, we used a non-fiber based method for optode placement, which while being restricted to measuring activation over the prefrontal cortex, is more comfortable and is associated with reliable skin contact. We compared laterality index, based on the lateralization patterns of the hemodynamic response, and OV Index across toddlers, and analyzed the relation between these measures and developmental ability. In sum, the combination of recording during audiovisual presentation of a children's show and using a relatively comfortable frontal fNIRS band were selected to improve data acquisition in toddlers and to reduce the motion artifact.

Because neurodevelopmental and language disorders are frequently associated with rightward or bilateral distribution of language-related activation compared to leftward lateralization in controls (Whitehouse and Bishop, 2008; Lindell and Hudry, 2013; Nielsen et al., 2014; Xu et al., 2015), we hypothesized that this relation could be detectable during audiovisual presentation of children's shows. We further hypothesized that lower developmental abilities, including language, would be associated with lower OV Index values. Finally, since language development is strongly correlated with more general cognitive development in toddlers (Oliver et al., 2004), we investigate whether these associations are specific to language or related more to general cognitive development in order to determine potential value of these metrics in serving as markers of risk for developmental delays.

## Materials and methods

This study was approved by an Institutional Review Board at the National Institutes of Health. Parents of all participants completed informed and written consent prior to their child's participation.

### Participants

Participants included 29 children (11 female) between the ages of 18 and 43 months, (mean = 29.57, *SD* = 7.18) with varying language abilities (see Table 1). Toddlers in this pilot study were recruited from either a typically developing group (*n* = 21, 7 female) or a language delay group (*n* = 8, 4 female), with the intent to follow children until they were 3 years of age to study aspects of development (NCT01339767) and early indicators of continued delays. Inclusion criteria for the typically developing group included T-scores>35 on all domains of the Mullen Scales of Early Learning (MSEL, Mullen, 1995) and no parent-reported history of delays. Inclusion criteria for the language delay group included expressive and receptive language scores in the “Very Low” range (T-score <30) on the MSEL at the time of screening, which occurred between 12 and 18 months of age. As expected, some children in the language delay group also scored below average on other aspects of cognitive development (see Table 1). Exclusion criteria for both groups included prematurity at birth, motor or other medical impairment deemed responsible for delays, and known genetic disorder. All children were recruited from the community through both advertisements and referrals from providers.

**Table 1:** Demographics.

	**Age in months Mean (SD) [range]**	**Sex (m:f)**	**Verbal AE^a^ Mean (SD) [range]**	**Non-verbal AE^a^ Mean (SD) [range]**	**Handedness^b^ (r:l:unknown)**
Total Sample	29.57 (7.18) [18.48–43.92]	18:11	29.25 (9.93) [6.5–45.5]	30.84 (9.09) [15–50]	20:3:6
Language Delay	30.28 (9.29) [19.32–43.92]	4:4	20.62 (10.34) [6.5–33]	25.62 (6.55) [15–32.5]	6:0:2

a *Verbal (Receptive and Expressive Language) and Non-verbal (Visual Reception and Fine Motor) Age Equivalents (in months) based on performance on the Mullen Scales of Early Learning*.

b *Handedness was assessed at the 36 month visit*.

### Procedures

The fNIRS session occurred at one of the regularly scheduled study visits, which occurred when the child was ~18, 24, or 36 months. We first attempted fNIRS only in children at 24 or 36 months, before trying to acquire data in children as young as 18 months. As part of a longer fNIRS session, Toddlers underwent a vanilla baseline recording, during which they watched two 50-s clips from children's shows, presented in audiovisual format. The vanilla baseline is a paradigm used during physiological data collection, such as EEG and ECG, in order to better homogenize participants' experience while maintaining attention throughout the data collection period (Jennings et al., 1992). Audiovisual clips from the Elmo's World segments from *Sesame Street* were chosen because they were engaging and maximized toddlers' attention while reducing motion artifact. While these videos were not designed to isolate particular functional abilities, they included Elmo interacting with children and animals through speech, gesture, and song. The videos (trials) were displayed on a 14-inch monitor placed at a distance of 40–60 cm from the participant. The video frame rate was at 29 frames per second and the audio sample rate was at 44 kHz. Children watched these videos after completing developmental and diagnostic assessments in a pediatric research clinic. Most of the younger toddlers (18–24 months) watched the videos while seated on a parent's lap, whereas most of the 36-month-olds watched the video while seated in a child-sized chair.

Handedness was determined at 36 months by systematic observation of dominance displayed on behavioral tasks (e.g., grasping pennies, throwing a ball). Non-verbal mental age was calculated as the mean of the age equivalents from the MSEL visual reception and fine motor subscales, and Verbal mental age was calculated as the mean of the age equivalents from the MSEL receptive language and expressive language subscales at the time of their fNIRS visit. Then, Non-verbal and Verbal Developmental Quotients (DQs) were calculated by dividing each toddler's age equivalent on the MSEL by their chronological age and multiplying by 100. Using DQ as an indicator of relative developmental status (compared to using T-scores) provides a measure reflecting the variability of the sample that is less influenced by floor effects. A Composite Developmental Quotient (Composite-DQ) was calculated based on the average of non-verbal and verbal developmental quotients.

### fNIRS

**Figure 1:**
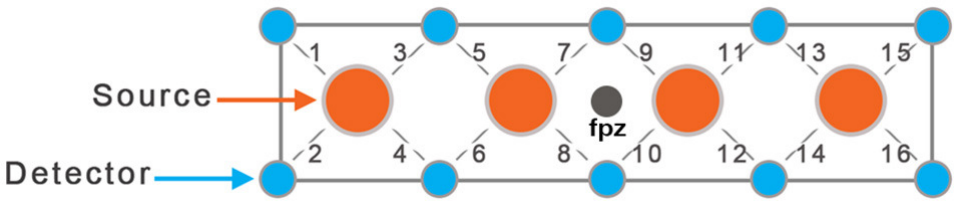
Schematic of fNIRS sensor with four sources and 10 detectors (16 source-detector pairs).

In this study, we used a continuous wave fNIRS system (fNIRS Devices LLC, MD). The instrument consists of an array of four sources and 10 detectors, with a total of 16 source-detector pairs (see Figure 1). The source-detector separation was set at 2.5 cm. While in fiber-based systems each source and detector has separate fiber connection, in this system all sources and detectors are molded together in a single silicon band. This non-fiber based sensor is portable and easier to apply on the forehead region (due to lack of hair) especially in toddler population. It collects data at two wavelengths—730 and 850 nm—with an acquisition frequency of 2 Hz. The sensor band was positioned on each child's forehead covering the prefrontal cortex (PFC). Due to a smaller head size in toddlers, only the middle channels (5–12) were used for data analysis. The sources and detectors were centered horizontally at FPZ based on the international 10–20 coordinate system (see Figure 1). This system was selected for this feasibility study because it is comfortable and easy to wear, portable, and inexpensive compared to fiber-based caps that cover the whole head, making this and similar systems potential candidates for use in early screening in infants and toddlers.

NIRS light intensities at two wavelengths were then converted to changes in oxy- and deoxy-hemoglobin. Here, the modified Beer-Lambert (MBLL) law was used for calculating changes in concentration of HbO and Hb. For MBLL, we used two differential pathlength factors (DPF) to account for two wavelengths and each subject's age (Scholkmann and Wolf, 2013). The DPFs for each age were calculated based on the following formula: DPF(λ,A) = α + βA^γ^ + δλ^3^ + ελ^2^ + ζλ, where A = age and λ = wavelength (e.g., DPF(730,2) = 5.4, DPF(850,2) = 4.32).

Processing of the raw NIRS signal involved detection and removal of artifacts related to subject motion as well as respiration and heart rate. We used both median filtering and the sliding window motion artifact rejection (SMAR) to detect and remove motion artifact and saturated channels (Ayaz et al., 2010). This algorithm uses presence of sharp spikes and high standard deviations of the signal (>3% temporally) to detect motion. It should be noted that while these filters were applied with the goal of removing any signal contaminated with motion artifact, the 29 children whose data is used here did not show significant motion artifact during the resting period. Thus, all data recorded during the audiovisual resting stimuli for these children were usable. Correlation Based Signal Improvement (CBSI) was also used to remove any unidirectional changes in Hb and HbO signal (Ayaz, 2010; Cui et al., 2010). This method is based on the assumption that there should be a negative correlation between HbO and Hb signal. The algorithm for CBSI is based on the linear combination of these two signals, resulting in an improved HbO signal that contains information from Hb signal. It should be noted that the processed HbO signal had been altered by Hb data patterns during the CBSI step. Then, a low pass frequency filter (<0.1 Hz, Hanning window, order 20) was applied to remove high frequency contamination related to heart beat and respiration (Izzetoglu et al., 2007; Kreplin and Fairclough, 2013; Naseer and Hong, 2013). To avoid edge artifacts, filtered data included all samples from 30 s prior to the trial to 30 s after trial completion. Afterward, signals were detrended (based on Linear detrending where the best straight-line fit from the signal is removed) to eliminate the slow drifts in the signal. Then, the HbO signals from the left channels and right channels were averaged separately and over the two trials, each including 50 s of data (i.e., 100 samples) to improve the signal to noise ratio. We used the HbO signal since it has been shown to be a better correlated of BOLD fMRI signal and have a better signal to noise ratio compared to Hb and has been commonly used in NIRS studies (Strangman et al., 2002b; Greve et al., 2009; Tong et al., 2011; Sato et al., 2013; Yue et al., 2013; Kawano et al., 2016). Hemodynamic response curves were detected using Matlab as an increase in HBO signal, followed by gradual decrease. We then calculated the laterality index based on the percentage difference between area under the curve (AUC) of the HbO signal for left and right prefrontal cortex, such that positive values indicate greater left vs. right activation, while negative values indicate higher right activation. The absolute value of the laterality index therefore provides the magnitude of difference between left and right activation.

Furthermore, we computed OV index for each child to quantify the observed hemodynamic oscillations in frequencies related to cerebral autoregulation (<0.1 Hz). OV index characterizes the level of variability of oxygen saturation at a given frequency band. First the instantaneous amplitudes of HbO and Hb data are calculated to quantify instantaneous oxygen saturation at the frequency band related to cerebral autoregulation. The instantaneous amplitude of each signal (*A*(*t*)) is calculated based on the analytic signal continuation approach (Boashash, 1992).

$$v\left(t\right)=S\left(t\right)+jH\left|S\left(t\right)\right|=A\left(t\right)e^{-j\varphi \left(t\right)}$$

Where *S*(*t*) is the real signal, *H*|*S*(*t*)| is the Hilbert transform of the signal, and v(t) indicates the complex signal in the time domain. We then calculate instantaneous oxygen saturation (*SO*_2_) based on a ratio of instantaneous amplitudes of changes in oxy-hemoglobin (HbO) to total hemoglobin (Hb+HbO) ($SO_{2}\;=\;\frac{HbO}{Hb+HbO}$). Therefore, in calculation of OV Index, both HbO and Hb have been taken into account. We defined OV index as the coefficient of variation (σ/μ, ratio of mean to standard deviation) of the instantaneous oxygen saturation signal (Anderson et al., 2014).

### Statistical analysis

Shapiro-Wilk normality tests were performed to test the normal distribution of both the OV index and Lateralization quotients. This test did not support non-normal distribution for either metric [Laterality index: *F*_(29)_ = 0.95, *p* = 0.27, OV index: *F*_(29)_ = 0.96, *p* = 0.42]. Moreover, there was no significant correlation between age and Laterality index or OV Index (*r* = −0.076, *p* = 0.35 and *r* = −0.096, *p* = 0.31, respectively). Therefore, all further analyses were collapsed across age. Then, we calculated the Pearson Correlation Coefficient values between the fNIRS measures (i.e., OV Index and Laterality index) and behavioral measures (i.e., Verbal, Non-Verbal and Composite Developmental Quotient) across all subjects.

## Results

### Toddler tolerance

As shown in Table 2, we successfully collected data during the audiovisual simulation from 29 out of 37 toddlers between the ages of 18–36 months with varying levels of language development. The percentages of successful NIRS data acquisition sessions were 80 and 76% for 18–24 months old and 36 months old toddlers, respectively. Further analysis using ANOVA showed no difference in Composite DQ for successful vs. unsuccessful fNIRS acquisitions [*F*_(1, 35)_ = 0.89, *p* = 0.352].

**Table 2:** Success rates and final sample size for fNIRS data acquisition in toddlers across two age ranges (18–24 and 36 months) with varying levels of language development.

	**18–24 months**	**36 months**	**Total sample size**
Attempts	20	17	37
Would not tolerate/wear the sensor	4	2	6
Excluded for excessive motion	–	2	2
Final Sample	16	13	29

### OV index

**Figure 2:**
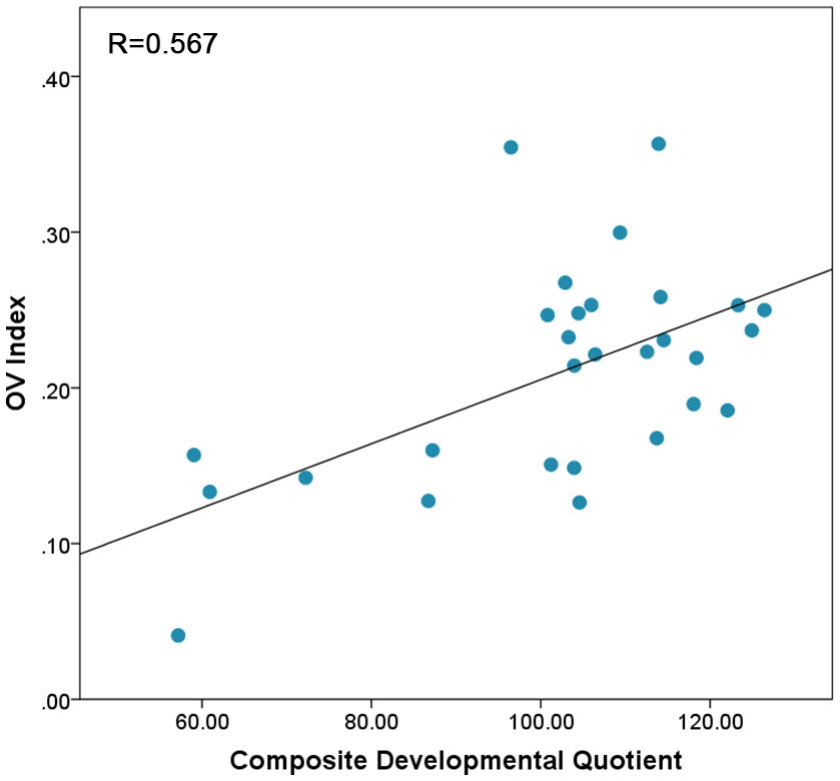
Correlation between OV index values combined across Left and Right prefrontal cortex and Composite-DQ across all toddlers exhibiting trend of higher OV index in toddlers with higher Composite-DQ.

There was no significant difference between the OV index from left and right PFC [*t*_(28)_ = −0.156, *p* = 0.87]. Therefore, the OV index from left and right were combined. We ran a two-tailed Pearson correlation between OV index and Composite Developmental Quotient (Composite-DQ), as well as verbal and non-verbal DQs. The result based on the combined OV index showed a significant correlation between OV index and Composite-DQ (*r* = 0.567, *p* = 0.001), Verbal DQ (*r* = 0.503, *p* = 0.005), and Non-Verbal-DQ (*r* = 0.53, *p* = 0.003). Toddlers with lower developmental scores showed a lower OV index (Figure 2).

In addition, OV index was correlated with the T-scores on the visual reception, receptive language, and expressive language subscales of the MSEL across all toddlers, with higher scores associated with a higher OV index (*r* = 0.542, *p* = 0.002; *r* = 0.449, *p* = 0.015; and *r* = 0.463 *p* = 0.011, respectively). OV index was not significantly correlated with fine or gross motor subscales (*r* = 0.35, *p* = 0.06 and *r* = −0.16, *p* = 0.53, respectively).

### Lateralization

**Figure 3:**
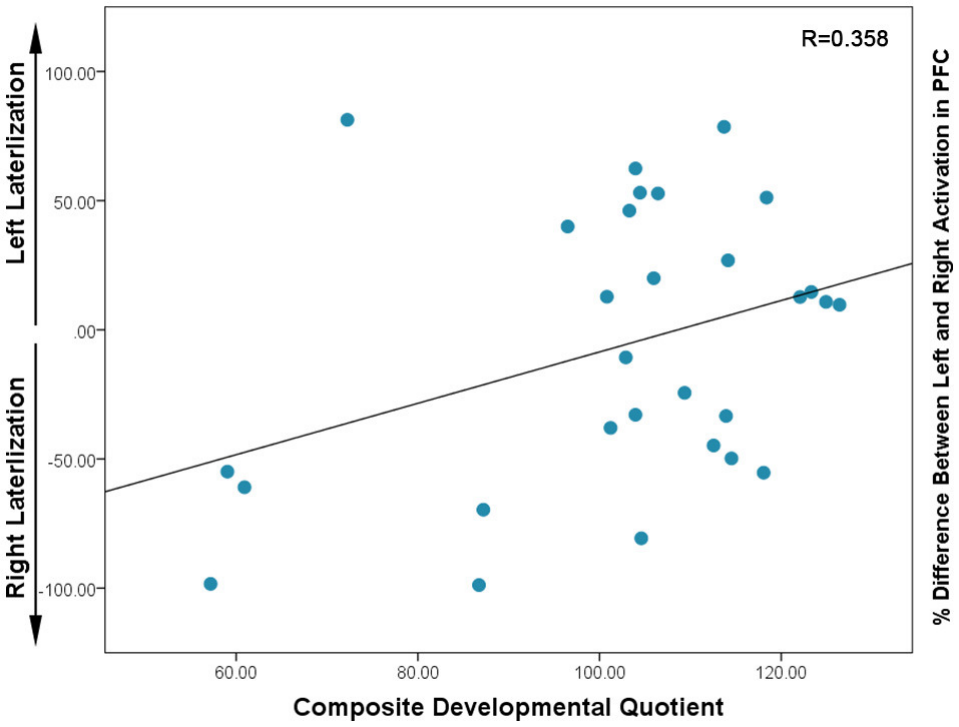
Correlation between Composite DQ and Laterality Index (based on percent difference between left and right activation) for each subject: negative values indicate right lateralization while positive values show left lateralization. Higher values in the positive and negative direction indicate the greater activation in left or right PFC, respectively.

**Figure 4:**
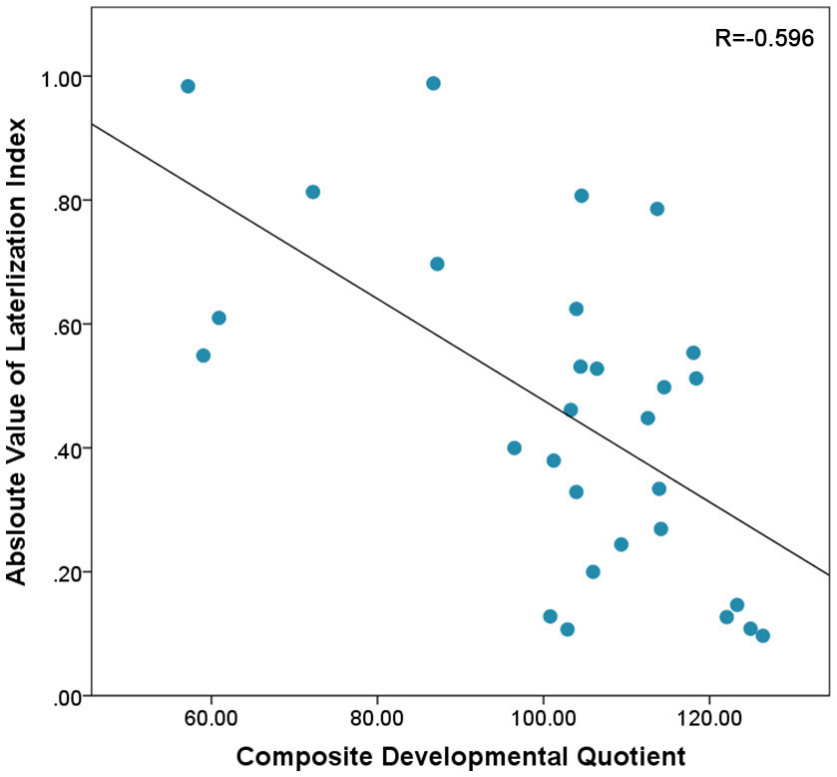
Correlation between Absloute values of Laterlization Index and Composite-DQ indicating higher differences between left and right PFC activation in subjects with lower Composite-DQ.

In this sample, there was a non-significant trend toward a positive correlation between Composite-DQ and laterality index (*r* = 0.358, *p* = 0.056). Specifically, toddlers with a lower Composite-DQ exhibited more rightward activation (Figure 3). There was no significant correlation between laterality index and Verbal or Non-Verbal DQ (*r* = 0.323, *p* = 0.088 and *r* = 0.324, *p* = 0.086). Moreover, there was a significant negative correlation between Composite-DQ and the absolute value of the laterality index (*r* = −0.596, *p* = 0.001). Specifically, toddlers with a lower Composite-DQ showed a greater discrepancy between left and right hemisphere activity (Figure 4). A similar pattern was found between Verbal and Non-Verbal-DQ and laterality index (*r* = −0.5, *p* = 0.006 and *r* = −0.6, *p* = 0.001).

Absolute value of the laterality index was negatively correlated with the fine motor, receptive language and expressive language T-scores on the MSEL (*r* = −0.627, *p* = 0.000; *r* = −0.436, *p* = 0.018; *r* = −0.501, *p* = 0.006). Specifically, toddlers with a larger difference between left and right activation patterns showed lower scores.

## Discussion

In this study, we examined hemodynamic response—via laterality index and OV index—in toddlers with varying levels of developmental ability. First, we show feasibility for use of a NIRS frontal band with toddlers, including those with language delays. Second, we found potential for the utility of both metrics as potential indicators of developmental risk. Specifically, toddlers with lower developmental scores showed lower OV index across hemispheres, as well as a pattern of greater differences in activation between hemispheres, along with a potential pattern of rightward activation. These early results suggest the feasibility of fNIRS as a potential modality to measure brain activity that may relate to neurodevelopmental differences in toddlers.

Toddlers in the present study generally tolerated the fNIRS headband and produced usable data. The percent of toddlers who produced usable data is similar to success rates seen in older children using fMRI (Yerys et al., 2009), including those toddlers with developmental delays. Future studies are necessary to determine if findings suggested in this feasibility study are indeed applicable to a larger population of toddlers.

We demonstrate preliminary evidence of an association between developmental ability and OV index within this paradigm. Here, OV index reflects the degree of oscillation in oxygen saturation within the frequency range associated with cerebral autoregulation. Our results are in accordance with previous studies of changes in cerebral autoregulation, cerebral blood flow, and OV index in children and adults (Chiron et al., 1992; Schoning and Hartig, 1996; Anderson et al., 2014; Chernomordik et al., 2016). Studies in children and adults have also indicated a relation between lower variability in autoregulatory responses with poor cognitive outcome (Vavilala et al., 2004; Silvestrini et al., 2006; Turalska et al., 2008). For example, relative degree of these oscillations and therefore magnitude of the OV index has been found to be lower in a Traumatic Brain Injury (TBI) population (Chernomordik et al., 2016). It is worth mentioning that the OV index is not a direct measure of cerebral autoregulation. Rather, it is associated with frequencies related to this mechanism and serves to quantify oscillations in those frequencies. The significance of these slower oscillations and their origin are still unknown. In addition, although we used the frequency cutoff of 0.1 Hz to reduce the effect of Mayer waves, this metric may be affected by Mayer waves because they share the spectral range with hemodynamic response. The effect of Mayer wave oscillation on HbO signal can also be more prominent in scalp regions and correction of the signal using shorter distance channels in future studies can be useful (Yucel et al., 2016). While this study suggests that some features of prefrontal hemodynamics may vary in toddlers at risk for developmental delays due to early language delay, more research in this age group is required to clarify the specificity of these differences.

Our finding of differential lateralization patterns as a potential correlate of general developmental delay is consistent with the extant literature. Lateralization has been shown to be a marker of abnormal development in previous studies, such as those using fMRI. Redcay et al. (Redcay and Courchesne, 2008) showed that in comparison to typically developing children, children with ASD recruited greater right hemisphere frontal lobe activity while listening to language sounds during sleep Those authors also found a positive correlation between language ability and right hemisphere activation in children with ASD, suggesting a compensatory role of right hemisphere regions in language processing in ASD. More recent research on children with language delay suggests at the population level, the lack of lateralization is a marker of risk for language impairment (Bishop et al., 2014). The present study was a feasibility study with a limited sample size; thus, replication and extension with longitudinal follow-up are required to clarify the role of left and right lateralization in cognitive development or language. With a larger sample size and more developmental variability, it will be useful to explore whether NIRS studies may be able to differentiate specific developmental problems (e.g., language delays, global developmental delay, or other delays). Correlation between absolute value of the laterality index and both verbal and non-verbal aspects of cognition indicates that group differences on this measure may be capturing the effects of general developmental delay rather than language delay, specifically. As such, the results of this study may reflect brain activity differences relating to general developmental abilities.

### Limitations

In addition to investigating whether prefrontal hemodynamic patterns can potentially signal presence of developmental delay, it will be important to determine if those patterns are also related to change in developmental status. Here, we initially attempted NIRS at 24 rather than 18 months, and only attempted at 18 months after accumulating evidence of the headwear being tolerated at 24 months. Non-etheless, simultaneous behavioral and neural measurement at 24 and 36 months allows for a more comprehensive profile to be explored. In addition, if these findings are replicated in larger studies in relation to developmental trajectories, it will also be essential to measure factors that may be mediating the relation between lateralization and group status (e.g., attention, autonomic functioning).

Another limitation of the present study is that we measured activation during audiovisual presentation of children's shows. Therefore, relating the findings of the study to specific cognitive functions is challenging, as participants were not engaged in a cognitive task during data collection. The results are therefore most useful in suggesting feasibility of fNIRS methodology in toddlers and for producing hypotheses for future work. For example, it is possible that toddlers with typical development processed the verbal aspects of the video very differently from toddlers with language delays who are at-risk for persistent developmental delays, and that this difference alone would explain observed differences in NIRS signal. Therefore, future research should investigate how the nature of stimuli present during acquisition (i.e., social, non-social, verbal) affects NIRS lateralization patterns and OV Index in children with typical and atypical development. Specifically, it will be important to compare resting state and event-related designs to systematically determine which best captures patterns of developmental variation and how this relates to any potential trade-offs with increased motion artifact in event-related designs. This is an essential next step in determining which design would be more practical for potential clinical use in toddlers.

The videos selected and number of trials used were chosen to maximize quality data acquisition in toddlers rather than characterizing functional responses to specific stimuli. While using this video and the given number of trials was associated with very high rates of toddlers tolerating the NIRS equipment and producing usable data, understanding of task related changes in the NIRS signal will require use of an increased number of more controlled stimuli. The field of neuroscience in the toddler age group is still new, and there are currently no published fNIRS studies in the age group of 18–36 months. As with the use of EEG in toddlers, the use of fNIRS in toddler neuroscience will likely involve shorter periods of data acquisition. Given the high temporal resolution of fNIRS, the duration of hemodynamic response can be captured within 12 s, (Huppert et al., 2006). In this study, we designed the stimuli length to obtain a continuous measure and ensure sufficient timing to include changes in hemodynamic response. Although currently there are no resting state data in 18–36 months old toddlers using fNIRS, the length of usable data in our study is within the range of studies in infants using EEG or NIRS. It is common for EEG studies in infants and toddlers to complete analyses with <1 min of data per participant (Friedrich and Friederici, 2005; Tierney et al., 2012; Jentschke et al., 2014; Gabard-Durnam et al., 2015). Similar timing has been used in event-related fNIRS studies of infants and toddlers (Baird et al., 2002; Nakato et al., 2009; Wilcox et al., 2012; Lloyd-Fox et al., 2014). As one of the main goals of our study design was to reduce the data loss in toddlers, the data sample used in our analysis is based on usable data that is sufficient for statistical analysis.

Another limitation of this study is lack of integration of handedness data with fNIRS lateralization patterns. This limitation is due in part to the fact that handedness is not fully established in toddlers and cannot be reliably assessed until age of four (Bryden et al., 2000; Scharoun and Bryden, 2014). Future longitudinal studies will be able to address the effect of handedness in early lateralization patterns.

The hemodynamic signal in fNIRS can also be contaminated with changes in blood flow in the skin, making it difficult to determine the strength of cerebral sources of oxygenation change. However, task-related effects on skin blood flow have been shown to be negligible (Mancini et al., 1994; Sato et al., 2013; Funane et al., 2014). Studies of hypercapnia, using a continuous wave system similar to the one used here, show that changes in the signal originate predominantly from the cerebrum rather than the skin (Themelis et al., 2007). In this study we use a source-detector separation that allows for differentiation of signals coming from the cerebrum vs. skin (Strangman et al., 2002a). However, it is also correct that group differences could be related to global neurophysiological differences, (i.e., increased blood flow generally related to attention and arousal) and comparing either specific cerebral regions or matched conditions can be helpful (Aslin and Mehler, 2005). For this reason, comparing activation between hemispheres becomes useful as individual differences in such a comparison would be more likely to be driven by local changes in cerebral hemodynamics.

Finally, the present study focuses on the prefrontal cortex. We selected a small, comfortable NIRS sensor band that takes advantages of the hairless skin on the forehead to achieve improved signal quality while maintaining comfort for toddlers. The fNIRS system is comfortable and affordable, with less potential for artifact compared to the fiber based system, especially for the toddler population, because of the larger surface area over which the optodes make consistent contact with the skin. Given that the development of the PFC and its cognitive function during early childhood is substantial and plays vital roles in cognitive and developmental abilities in children (Kwon et al., 2002; Hare and Casey, 2005; Davidson et al., 2006; Durston et al., 2006; Casey et al., 2008; Tsujimoto, 2008) studying the PFC may be useful in the toddler population. In the context of this study, we examined if we could detect developmental variations within the prefrontal cortex region with possible important implication of using fNIRS in clinical setting. While the PFC plays a major role in language and social processing, other important regions of interest (e.g., temporal language areas, parietal association areas) could not be studied with the current sensor design. This study, however, is useful in suggesting that differences in activation can be captured within the prefrontal cortex, and that those differences could therefore potentially be a correlate of risk. As such, while the present study describes measures of frontal cortex hemodynamic patterns that may be useful in detecting early delays, it cannot provide a measure of functional brain activation related to language and communication.

## Conclusion

Overall, the results of this study provide evidence for feasibility of the use of fNIRS methodology in toddlers, including the 18–36 months age range as well as in toddlers with varying levels of language development. Further, preliminary results suggest that decreases in OV index and larger lateralization differences in toddlers are associated with specific measures of lower developmental ability. Future studies with longitudinal designs, controlled stimuli, and larger and more diverse subject populations will be necessary to determine the role of prefrontal cortical hemodynamics as potential biomarker for neurodevelopmental disorders.

## Author contributions

AA wrote and revised the manuscript and prepared the figures and tables, and performed data analysis; ES revised the manuscript, performed behavioral analysis, and task design and prepared the tables; ES, FC, and AT, edited manuscript AA and EC. Revised the manuscript FA advised regarding the analytical concepts ES and FC performed data acquisition; ES, AA, AT, and SM contributed to the study design; BS and LS. Performed behavioral assessment; AT, DM, and AG gave technical support and conceptual advice AT and AG supervised the study and reviewed manuscript AG supervised the analysis and provided analytical insight.

### Conflict of interest statement

The authors declare that the research was conducted in the absence of any commercial or financial relationships that could be construed as a potential conflict of interest.
